# The Open Algorithm Based on Numbers (ABN) Method: An Effective Instructional Approach to Domain-Specific Precursors of Arithmetic Development

**DOI:** 10.3389/fpsyg.2018.01811

**Published:** 2018-09-25

**Authors:** Gamal Cerda, Estíbaliz Aragón, Carlos Pérez, José I. Navarro, Manuel Aguilar

**Affiliations:** ^1^Facultad de Educación, Universidad de Concepción, Concepción, Chile; ^2^Departamento de Psicología, Universidad de Cádiz, Cádiz, Spain; ^3^Dirección de Pregrado, Universidad de O’Higgins, Rancagua, Chile

**Keywords:** domain-specific precursors, TEMA-3, early arithmetic, ABN method, mathematics

## Abstract

This article presents the results of a comparative study regarding the impact and contribution of two instructional approaches to formal and informal mathematical reasoning with two groups of Spanish students, aged four and five. Data indicated that for both age groups, children under the ABN method [Open Algorithm Based on Numbers (ABN)] (*n* = 147) achieved better results than the group under the CBC approach (Closed Algorithms Based on Ciphers) (*n* = 82), which is the widespread approach in Spanish schools to teach formal and informal mathematical reasoning. Furthermore, the comparative analyses showed that the effect is higher in the group of students who received more instruction on skills considered domain-specific predictors of later arithmetic performance. Statistically significant differences were found in 9 of the 10 dimensions evaluated by TEMA-3 (*p* < 0.01), as well as on estimation tasks in the number-line for the 5-year-old-group. However, the 4-year-old group only presented significant results in calculation and concepts tasks about informal mathematical reasoning. We discuss that these differences arise by differential exposure to specific number-sense tasks, since the groups proved to be equivalent in terms of receptive vocabulary, processing speed, and working memory. The educational consequences of these results were also analyzed.

## Introduction

During the 1st years of their lives, students pay special attention to their environment and innately show curiosity about the quantitative relationships that occur around them, thus developing informal mathematical reasoning. These skills are the basis for the mathematical concepts taught at school. As students begin receiving formal instruction, mathematical reasoning is developed and refined (Ginsburg et al., 1998). Children leave aside intuition and develop different types of arithmetic reasoning, such as algebraic reasoning and verbal reasoning, among others. Formal mathematical reasoning requires from students a competent level in the management of symbols and language (Godino and Font, 2003). In particular, formal mathematical reasoning involves conventional knowledge related to number literacy as well as knowledge about the basic concepts of the decimal system. Furthermore, it also includes the knowledge of number facts and calculation, since instruction and memorization are necessary both for the recovery of number facts as well as for carrying out complex arithmetic operations, such as addition and subtraction with regrouping, solving operations with numbers with middle-zeros, etc. (Robinson et al., 2018).

In order students attain the necessary arithmetic skills for the curriculum content acquisition, it is necessary to ensure they develop from an early age, and in an up-close and meaningful way, contents such as counting as well as relational aspects and processes: problem-solving and number representation (Ginsburg and Baroody, 2007; Alsina, 2012). This is the reason why researchers are focusing their efforts on the study of the mechanisms underlying the arithmetic skills development. One of the main goals is the identification and analysis of the predictor variables for arithmetic performance. They are variables on which the most complex mathematic learning is developed (Cargnelutti et al., 2017; Cerda et al., 2015).

There is a long researching studies that defends the role of number sense as a strong predictor of successful mathematics performance, above other general factors such as vocabulary and working memory (Jordan et al., 2007, 2008, 2009). Jordan et al. (2008) define the *number sense* as the ability to understand numbers and arithmetic operations, together with the capacity to make arithmetical judgments resulting from the understanding of numbers and arithmetic facts. According to Jordan et al. (2008), number sense includes the ability to count, knowledge of numbers, and number facts. The topic linking the role of number sense and performance in mathematics is also supported because in having a weak number sense, makes the formal instruction process more difficult for students. This difficulty continues when students go along the compulsory schooling (Baroody and Rosu, 2006). In addition, there is also substantial evidence that accounts the significant relationship between cardinality and number series understanding, achievement of multiplication solving problems, addition and subtraction (De Smedt et al., 2013; Lyons et al., 2014; Vogel et al., 2015).

Counting could also be an important predictor of mathematical performance (Geary, 2011; Bartelet et al., 2014). So, number knowledge involves recognizing differences between quantities and making judgments about the identified quantities. Although younger children rely on visual perception instead of counting to make these type of judgments, however, it has been found that at the age of six, most children incorporate notions of quantities and counting schemes into a mental number-line (Siegler and Booth, 2004). Children associate that numbers presented after other numbers in a counting sequence and, therefore, in a number-line, correspond to a higher number than those presented before. Consequently, students develop a linear representation of numerical magnitudes, which support the learning of the positional value of numbers and the elaboration of mental calculation. This linear representation, along with the operation of counting, should predice mathematics learning difficulties (MLD) (Geary et al., 2009). Cirino (2011) also identified the symbolic and non-symbolic comparison tasks, and the principles or concepts related to competent counting, such as symbolic labeling and knowledge of the number sequence, as latent variables related to number knowledge.

It has also been maintained that infants can make computation using physical references (Jordan et al., 2008). However, as children start formal education, they begin to use algorithms, understood as sequences of unambiguous instructions used to obtain a required result (Jordan et al., 2008). These sequences contribute to understand some basic mathematical concepts, such as seriations, patterns, forms, comparisons, estimations, verifications, etc. (Levitin, 2015). Key factors associated with the number concept emerge as precursors of mathematical development, and improve the understanding of fundamental arithmetic concepts and facts that, with appropriated instructional approaches, should enable children a higher academic achievement.

In general, teaching methods in formal education are facilitated by the use of textbooks, designed to meet the curriculum needs. These books offer an organized scheme for teaching and learning (Fan et al., 2013; Hadar, 2017). Within the Spanish and international context of mathematics formal instruction, (even if there are differences attributable to teacher’s management, textbooks, and schools’ pedagogical guidelines), there is a certain homogeneity in terms of teaching methods. Namely, the most extended mathematics teaching approach focuses on learning of additive and multiplicative structures through algorithmic processes based on number figures (Barba-Uriach and Calvo, 2012). We will refer to this approach as the Closed Algorithms Based on Ciphers (CBC) methods.

The algorithms from the CBC methods develop after understanding the numbers’ place value. CBC is implemented with numbers. The facts numbers, sequence of steps, and calculations required by the process (which determine the arithmetic tasks), are completely predefined when the quantities involved in the algorithmic are established. In this sense, if two individuals use the same algorithm to perform an arithmetic task, both subjects will necessarily follow the same intermediate steps. Moreover, in a traditional CBC approach, the partial calculations completed is not a requirement to the problem that is being solved. Thus, they become only crucial steps for the correct functioning of the algorithm. In addition, in a CBC approach, there is more room for error, because non-specific reference mechanisms to monitor the problem’s partial steps are not trained.

Faced with this condition, several Spanish countries school systems are currently implementing the Open Algorithm Based on Numbers (ABN) method (Martínez-Montero and Sánchez, 2013). This approach gathers and addresses the natural route of each stages of the exploratory process that children utilize to understand numbers and their properties. This route is used as the background to design the instructional sequences and significant algorithms to perform facts with numbers. The ABN method has precedents in some educational proposals launched in the Netherlands to renew the mathematics teaching and learning in general and particularly teaching methodology for calculation. This was called “realistic mathematics.” This is oriented to development mathematical competence and fostering mathematical reasoning through manipulative and stimulating instruments for students in order to increase motivation and attention.

Implementation of ABN procedure begins in the academic course of 2008/2009 in a group of first grade of primary school in a public school at Cadiz (Spain). One year later, ABN extends in 4 more schools in the same province with approximate 125 students first graders. During the 2011–2012 different nationwide schools started implementing ABN method, distributed by more than 10 Spanish autonomous communities. Throughout 2013 keep growing the Spanish public schools using this method, and starting from the 1st year of preprimary education. In addition ABN begins to expand internationally in other countries such as Mexico, Argentina or Chile. But there are still no published results on these experiences. According to data provided by Cantos (2016) currently between 6,000 and 7,000 classrooms follow the ABN methodology, representing an approximate total number of 200,000 students learning math with ABN.

The integration of this path to the ABN method also provides an adequate understanding of the different steps for calculation process. Thus, with regard to the additive and multiplicative algorithmic, these processes can be understood as a transformation of resulting quantities from a dynamic operative process. Namely, a process in which each sequence is a sub-process involving explicit quantities, which are operated under the user’s criteria. Starting in early childhood, the ABN method focuses on performing math tasks with whole numbers, according to ranges or universes numbers. The method uses different formats for representation and manipulation of specific numbers, according to concrete, pictorial, abstract (CPA) instructional approach to teaching, that develops a deep and sustainable understanding of maths in pupils (Bruner, 1966). The principle of the ABN method is to maintain the numerosity of quantities all the times, in terms of knowledge, composition and decomposition, as well as taking into consideration how they operate in relation to other quantities (Martínez-Montero, 2010, 2011). In recent years, there has been evidence of the effect that this new methodology should have: to learn mathematics in a realistic and quotidian way, moving away from the mere acquisition of strategies and knowledge of symbols, that does not guarantee a realistic understanding (Bracho-López et al., 2014a). In this way, the unidirectional and sequential transfer of information is avoided, to give way to a more active learning process, in accordance with the context of student development (Novo et al., 2017).

With ABN approach, algorithmic are latent parts in the process of structuring mathematical knowledge. A meaningful mathematical understanding is developed throughout schooling. This knowledge structure also contributes to early approaches to Algebra, by experimentally incorporating mathematical concepts such as equations, powers, roots, etc. ABN approach requires focusing on the early skills acquisition to allow adequate understanding of the number but not only to reel number sequences off by memory. Thus, the ABN method focuses on developing in children the ability to establish differences or similarities between groups; relations between objects by grouping them according to specific criteria; pairing set elements with only one element from a different set; to intuit the order of objects according to number ranges; and, to use acquired problem-solving skills to elucidate daily life problems that involve counting. The flexibility that characterizes the ABN method not only offers an advantage in the development of original solution approaches or different types of solutions, but it also provides a set of strategies to adequately solve mathematic tasks (Torbeyns et al., 2005).

This previous background allows us to claim that a timely and adequate assessment of informal and formal reasoning (both regarding skills and concepts associated with early mathematical competence), can significantly contribute to the analysis and prediction of students achieving. Both dimensions are precursors of students’ performance in mathematics. In the same way, given the emphasis of the ABN method in working with quantities from the early stages, it is relevant to examine whether or not the exposure to a specific instructional approach for the development of mathematics skills, concepts and principles in early childhood education produces a differential level of development in students. In short, this research aims to determine potential differences linked to a specific instructional approach to formal and informal mathematical reasoning in a group of preschool children.

The following three hypotheses are proposed for this study:

(1) There are significant differences for ABN method students group in comparison to CBC group, in informal mathematical reasoning. Specifically in counting, comparisons, calculation and informal concepts, regardless of the school year.
(2) There are significant differences for ABN method students group in comparison to CBC group, in the formal mathematical reasoning. Specifically in number facts, formal calculation, and formal mathematical concepts, regardless of the school year.
(3) There are significant differences for ABN method students group in comparison to CBC group, in number-line estimation, regardless of the school year.

## Materials and Methods

A quasi-experimental descriptive and comparative cross-sectional design was used. Measurements of the dependent variables were taken in a single moment. Mathematical performance was compared in two different ages groups of students (4 and 5 years), with two types of mathematical instruction approaches (ABN and CBC). Considering the characteristics of the cross-sectional designs, three measures of control over cognitive variables were used in order to guarantee the groups’ equivalence and comparability. The designs of causal type, with control variables such as those used in this work, allow to establish some relevant inferences, given that the cause-effect relationships already occurred or occur during the process of measuring (Hernández et al., 2014). These kinds of designs have been used for that purpose in other similar qualified investigations (Wang et al., 2016).

### Participants

The total sample of students (*n* = 224) belonged to five public schools from Spain, four of them located in the community of Andalusia and one of them in the community of Madrid. In the Spanish public school system, most of the parent’s choice the school according with the standard of proximity to their homes. This criterion maintains the patterns of social stratification associated with residential zone (Mancebon-Torrubia and Perez Ximenez-de-Embun, 2014). Social and economic neighborhood similarities are usually coincident with the social and economic structure of schools settled on the same neighborhood. According to the OECD (2015) report, Spanish school system holds one of the highest social-economical homogeneity rates in classrooms. In this study, schools were settled in middle-class neighborhoods, considering that social-economic differences between students were not relevant. Teachers’ background was also similar, considering that in the Spanish education system all Pre-school and Primary school teachers hold a University degree. Thus, the socioeconomic composition of these five public schools corresponds to middle-class standards. The participants were 224 students, of which 110 (49.1%) girls and 114 boys (50.9%). The average age for the female group was of 65.47 months (*SD* = 6.98). The average age for the male group was of 64.33 months (*SD* = 6.45). 111 students belonged to the 4-year-old pre-school class, and 113 to the 5-year-old pre-school class. Although so far ABN is being used from 4 to 14 years old students, we consider that the beginning of the schooling and the first contact for children with the formal academic math is important for learning this subject. Several longitudinal studies shown that when young children start having trouble with mathematic, they keep on this problem later (Navarro et al., 2012).

Students taught under the Open Algorithm Based on Numbers method (ABN group) were 142; 74 aged 4, and 68 aged 5. Student under the Closed Algorithms Based on Ciphers (CBC group) were 82; 37 aged 4 and 45 aged 5. Students with special educational needs were not included in this study.

### Procedure

Trained professionals carried out participants’ assessment in two sessions. Each was of approximately 15–20 min. The purpose of this design was to attend to the particular characteristics of the students, and to avoid student’s tiredness. The evaluation conditions were optimal. The assessment was conducted in settings free of distractions that could interfere in the results. One session consisted in the administration of TEMA-3 test to evaluate students’ mathematical competence. The other session focused on the evaluation of the control variables (verbal working memory, receptive vocabulary, and processing speed). During this session, the number-line estimation test was also administered. Harvey and Miller (2017) reported that receptive vocabulary significantly affects to early math skills. Also, Peng et al. (2016) considered that processing speed and working memory as variables related to mathematical competence. These two subtests were used as control tests to establish the equivalence of the groups.

This study considered two groups of students, which will be referred to CBC-group and ABN-group. The CBC-group consisted of students who received instruction under the CBC approach. This type of education is widespread in most Spanish and other countries schools, and it characterizes for adjusting to the contents required by the educational administration of the country. CBC methodology focuses on the monitoring of content learning through textbooks. The didactic proposal of textbooks is mostly oriented to a CBC additive and multiplicative structure. The ABN-group was composed of students who received mathematical instruction through the ABN method. The teachers in charge of this group had a specific training in the ABN instructional method. Teachers’ training took into account the contents, competencies and specific goals required by the educational administration government for each grade. Both groups of participants received the compulsory mathematics contents stated in the school curriculum for each grade, but with different approach. Instructional timing was the same for both groups and it was accordingly to the instructional schedule established by the Spanish Ministry of Education. Thus, the significant difference between both groups was the mathematics instructional approach used. It is important to note that all participating students received mathematics instruction through one method or the other from the 1st year of preschool education.

All subjects gave written informed consent in accordance with the Declaration of Helsinki. Informed consent was obtained from parents, teachers and school principals involved in this study.

### Instruments

#### Test of Early Mathematics Ability-Third Edition, TEMA-3 (Ginsburg and Baroody, 2007)

This test assesses mathematical competence and consists of two subtests that focus on the evaluation of informal and formal reasoning, both in terms of skills and concepts. The informal reasoning subtest is composed of tasks aimed at the assessment of counting, comparison of quantities, informal calculation and basic informal concepts. The formal reasoning subtest evaluates conventions related to number quantity literacy, knowledge of number facts, formal calculation, and formal mathematical concepts.

TEMA-3’s administration was individual taking around 30 min. Administration timing differs according to the student’s age. The test is applicable to children aged between 3 and 9 years old. TEMA-3 has 72 items presented in order of increasing difficulty. The Cronbach’s alpha for this test was 0.91 for 4-year-olds and 0.95 for 5-year-olds.

In the Spanish standardization for TEMA-3, it is reported the following scores: 4 years (*M* = 15.25, *SD* = 5.89), and 5 years (*M* = 25.03, *SD* = 7.23). The range data (minimum and maximum score) do not appear in the Spanish standardization manual.

#### Numerical Estimation Task (Siegler and Booth, 2004)

This pencil-and-paper test evaluates estimation skills in a number-line. For its administration, participants are presented with a sheet of paper with a 20-centimeter number line, which starts at zero and ends at 20. Above the line, in the upper central part of the sheet, a number is shown. Participant must point out the number in the straight line. The test consists of 10 items, which correspond to the following numbers: 2, 4, 7, 8, 11, 13, 16, 17, 18, and 19, randomly presented. The mean comparison rate was calculated according to the number of correct answers with respect to the number requested by the test versus the number provided by the student. Answers were considered correct if they did not present a rate of error higher than ±15% for the requested number. The Cronbach’s alpha for this test was 0.80

#### Coding Subtest From the Wechsler Preschool and Primary Scale of Intelligence, Third Edition (WPPSI-III) (Wechsler, 2009)

This test is included within the Wechsler Intelligence Scale for preschool and primary school (Wechsler, 2009). It assesses processing speed, visual perception, visual-manual coordination, short-term memory, learning ability, and cognitive flexibility. The student must complete a set of 64 figures presented with the appropriate symbols. Participant must follow the reference models within a time limit of 2 min. The Cronbach’s alpha for this test was 0.84.

#### Receptive Vocabulary Test From the Dyslexia Screening Test - Junior (DST-J) (Fawcett and Nicolson, 2013)

This test is a measure of vocabulary mastery and reasoning ability. The purpose of this test is to evaluate receptive vocabulary through a multiple-choice format. The test comprises 18 items; each correct item receives one point. The Cronbach’s alpha for this test was 0.74.

#### Backward Digit Task From the Dyslexia Screening Test - Junior (DST-J) (Fawcett and Nicolson, 2013)

This test measures verbal working memory. It involves the oral repetition of digits in reverse order. As the number of trials increases, the number of digits increases and, consequently, the difficulty of the task. This task is composed of seven series of two items each. The test includes three items plus two additional, administrated in case that child has difficulties in properly understanding the instructions. The Cronbach’s alpha for this test was 0.85

### Statistical Analyses

In order to calculate the comparative analyzes between average scores obtained by the ABN and CBC groups, one-way ANOVA tests were completed. Whenever the homoscedasticity of the variances was not proven, a correction of the degrees of freedom, and Welch’s robust test was applied. The effect size was also calculated for the total variables measured.

## Results

In order to establish that the ABN and CBC groups were equivalent, three control tests were computed. A receptive vocabulary test, a processing speed test, and a working memory test of backward digits.

**Table 1** shows the correlations matrix of the scores and total scores of the subtests of formal and informal mathematical thinking of the TEMA-3 test reached by the students, with the purpose of analyzing the intensity of the associations between them. It is observed that all of them are statistically significant.

**Table 1:** Correlation matrix of the student scores in formal and informal mathematical thinking subtest of TEMA-3.

	Informal reasoning	Counting	Comparing	Informal calculations	Informal concepts	Formal reasoning	Conventions	Number facts	Formal calculations	Concepts
Informal reasoning	1	0.967^∗∗^	0.793^∗∗^	0.854^∗∗^	0.658^∗∗^	0.853^∗∗^	0.856^∗∗^	0.476^∗∗^	0.277^∗∗^	0.442^∗∗^
Counting		1	0.677^∗∗^	0.747^∗∗^	0.573^∗∗^	0.860^∗∗^	0.876^∗∗^	0.470^∗∗^	0.263^∗∗^	0.402^∗∗^
Comparing			1	0.662^∗∗^	0.466^∗∗^	0.638^∗∗^	0.643^∗∗^	0.323^∗∗^	0.218^∗∗^	0.376^∗∗^
Informal calculations				1	0.537^∗∗^	0.676^∗∗^	0.680^∗∗^	0.354^∗∗^	0.198^∗∗^	0.403^∗∗^
Informal concepts					1	0.546^∗∗^	0.464^∗∗^	0.472^∗∗^	0.309^∗∗^	0.345^∗∗^
Formal reasoning						1	0.944^∗∗^	0.692^∗∗^	0.476^∗∗^	0.489^∗∗^
Conventions							1	0.452^∗∗^	0.261^∗∗^	0.367^∗∗^
Number facts								1	0.622^∗∗^	0.253^∗∗^
Formal calculations									1	0.192^∗∗^
Concepts										1


**p < 0.01.

In order to examine if there were differences in the scores of these variables according to the age group, comparisons were made by means of simple ANOVA tests.

No significant differences were found in the receptive vocabulary test results for the 4-year-old group [*Mdn*_CBC_ = 11.56, *SD*_CBC_ = 1.96; *Mdn*_ABN_ = 12.17, *SD*_ABN_ = 1.82; *F*(1,109) = 2.604, *p* > 0.01]. Likewise, no significant differences were found for the WPPSI [*Mdn*_CBC_ = 27.02, *SD*_CBC_ = 11.20; *Mdn*_ABN_ = 26.79, *SD*_ABN_ = 11.03; *F*(1,109) = 0.011, *p* > 0.01] and neither were there significant differences in the comparison between the backward digit test for the 4-year-old group [*Mdn*_CBC_ = 1.48, *SD*_CBC_ = 1.30; *Mdn*_ABN_ = 1.63, *SD*_ABN_ = 1.47; *F*(1,109) = 0.251, *p* > 0.01].

Similarly, no significant differences were found in the receptive vocabulary test results for to the 5-year-old group [*Mdn*_CBC_ = 12.57, *SD*_CBC_ = 1.58; *Mdn*_ABN_ = 12.67, *SD*_ABN_ = 1.38; *F*(1,111) = 0.122, *p* > 0.01]. Likewise, no significant differences were found in the results for the WPPSI [*Mdn*_CBC_ = 34.80, *SD*_CBC_ = 9.43; *Mdn*_ABN_ = 35.64, *SD*_ABN_ = 10.43; *F*(1,111) = 0.192, *p* > 0.01]; and neither were there significant differences in the comparison between the backward digit test for the 5-year-old group [*Mdn*_CBC_ = 2.22, *SD*_CBC_ = 1.44; *Mdn*_ABN_ = 2.91, *SD*_ABN_ = 1.33; *F*(1,111) = 6.765, *p* > 0.01].

Several comparative analyzes were carried out in order to guarantee equivalence and comparability among ABN and CBC groups (gender, mathematical instructional method and autonomous community): *(a) Gender (4-year-old)*. For the 4 years aged group, vocabulary [*t*_(109)_ = 0.267; *p* > 0.05], processing speed [*t*_(109)_ = -2.314; *p* > 0.05], and working memory [*t*_(109)_ = -0.336; *p* > 0.05] differences between groups were not significant for gender; *(b) Mathematical instructional method (4-year-old)*. In the same way, there were no statistically significant differences in vocabulary [*t*_(109)_ = -1.614; *p* > 0.05], processing speed [*t*_(109)_ = 0.103; *p* > 0.05], and working memory [*t*_(109)_ = -0.501; *p* > 0.05] for math instructional method used; *(c) Autonomous community (4-year-old)*. Vocabulary [*t*_(109)_ = -0.475; *p* > 0.05], processing speed [*t*_(109)_ = 1.784; *p* > 0.05], and working memory [*t*_(109)_ = 0.351; *p* > 0.05] differences between groups were not significant for autonomous community of the schools where students attended; *(d) Gender (5-year-old)*. For the 5 years aged group, vocabulary [*t*_(109)_ = 0.267; *p* > 0.05], processing speed [*t*_(109)_ = -2.314; *p* > 0.05], and working memory [*t*_(109)_ = -0.336; *p* > 0.05] differences between groups were not significant for gender; *(e) Mathematical instructional method (5-year-old)*. In the same way, there were not statistically significant differences in vocabulary [*t*_(109)_ = -1.614; *p* > 0.05], processing speed [*t*_(109)_ = 0.103; *p* > 0.05], and working memory [*t*_(109)_ = -0.501, *p* > 0.05] for method of instruction; *(f) Autonomous community (5-year-old)*. Either, in vocabulary [*t*_(109)_ = -0.475; *p* > 0.05], processing speed [*t*_(109)_ = 1.784; *p* > 0.05], and working memory [*t*_(109)_ = 0.351; *p* > 0.05] for autonomous community of the schools where students attended, no statistically significant differences were found.

**Table 2** shows TEMA-3 subtests scores comparing the two instructional methods (CBC and ABN).

**Table 2:** Descriptive statistics for the different TEMA-3 test dimensions for the 4-year-old CBC and ABN groups.

Dimensions	Group	N	*M*	*SD*	CI 95%	MIN	MAX	*F*	η^2^
Informal reasoning	CBC	37	17.35	4.87	15.72–18.98	8	27	3.428	0.030
	ABN	79	19.18	6.03	17.82–20.53	4	35		
	Total	116	18.59	5.73	17.54–19.65	4	35		
Counting	CBC	37	11.22	2.95	10.23–12.20	6	19	2.392	0.021
	ABN	79	12.16	3.67	11.34–12.99	2	22		
	Total	116	11.86	3.47	11.22–12.50	2	22		
Comparison	CBC	37	2.51	1.07	2.15–2.87	1	5	0.486	0.004
	ABN	79	2.62	1.19	2.35–2.89	1	5		
	Total	116	2.59	1.15	2.37–2.80	1	5		
Informal calculation	CBC	37	2.03	1.30	1.59–2.46	0	4	4.154*	0.037
	ABN	79	2.52	1.35	2.21–2.82	0	5		
	Total	116	2.36	1.35	2.11–2.61	0	5		
Informal concepts	CBC	37	1.59	0.60	1.39–1.79	0	2	4.885*	0.043
	ABN	79	1.81	0.60	1.67–1.94	0	3		
	Total	116	1.74	0.61	1.62–1.85	0	3		
Formal reasoning	CBC	37	3.00	1.22	2.59–3.41	1	6	1.302 (*FWelch*)	0.009
	ABN	79	3.30	1.88	2.88–3.72	0	10		
	Total	116	3.21	1.70	2.89–3.52	0	10		
Conventions	CBC	37	2.08	1.14	1.70–2.46	0	5	1.345 (*FWelch*)	0.010
	ABN	79	2.35	1.52	2.01–2.69	0	6		
	Total	116	2.27	1.41	2.00–2.53	0	6		
Number facts	CBC	37	0	0	0	0	0	1.842	0.017
	ABN	79	0.07	0.30	0.0–0.14	0	2		
	Total	116	0.05	0.25	0.0–0.09	0	2		
Formal calculation	CBC	37	0	0	0	0	0		
	ABN	79	0	0	0	0	0		
	Total	116	0	0	0	0	0		
Concepts	CBC	37	0.92	0.28	0.83–1.01	0	1	0.165	0.002
	ABN	79	0.89	0.35	0.82–0.97	0	2		
	Total	116	0.90	0.33	0.84–0.96	0	2		
TEMA-3 Total score	CBC	37	20.35	5.84	18.40–22.30	11	33	2.968	0.027
	ABN	79	22.48	7.60	20.77–24.18	4	45		
	Total	116	21.80	7.13	20.49–23.11	4	45		


*p < 0.05.

Statistically significant differences were found (*p* < 0.05) between the CBC and ABN groups in informal calculation and informal concepts dimensions, although effect sizes were small. In formal reasoning calculations dimension, 4-year-old children were not able to correctly solve any task. A possible explanation should be, because the suspension criterion was used before being able to solve them. The same works to number facts dimension, where 4-year-old students did not solve any tasks. However, some ABN-group participants appropriately solved up to two tasks of this type (**Table 3**).

**Table 3:** ANOVA and effect size results of TEMA-3 dimensions for the 5-year-old CBC and ABN groups.

Dimensions	Group	N	*M*	CI 95%	MIN	MAX	*F*	η^2^
Informal reasoning	CBC	45	20.69	19.17–22.21	5	32	51.58**	0.317
	ABN	68	27.63	26.41–28.85	14	36		
	Total	113	24.87	23.73–26.00	5	36		
Counting	CBC	45	12.71	11.71–13.71	4	21	51.02**	0.315
	ABN	68	17.40	16.55–18.24	9	22		
	Total	113	15.53	14.76–16.30	4	22		
Comparing	CBC	45	3.22	2.92–3.52	1	5	15.25**	0.121
	ABN	68	3.99	3.73–4.23	1	6		
	Total	113	3.68	3.48–3.88	1	6		
Informal calculations	CBC	45	2.98	2.60–3.35	0	5	21.33**	0.161
	ABN	68	3.94	3.70–4.18	1	5		
	Total	113	3.56	3.33–3.78	0	5		
Informal concepts	CBC	45	1.82	1.66–1.98	0	3	19.29**	0.148
	ABN	68	2.29	2.15–2.43	1	4		
	Total	113	2.11	1.99–2.22	0	4		
Formal reasoning	CBC	45	3.58	3.01–4.14	0	12	40.69**	0.268
	ABN	68	5.97	5.48–6.45	2	12		
	Total	113	5.02	4.59–5.44	0	12		
Conventions	CBC	45	2.53	2.12–2.95	0	7	56.74**	0.338
	ABN	68	4.43	4.12–4.73	1	7		
	Total	113	3.67	3.37–3.97	0	7		
Number facts	CBC	0.08	0.46	-0.05–0.22	0	3	7.13** (*FWelch)*	0.049
	ABN	0.42	0.86	0.21–0.63	0	4		
	Total	0.29	0.75	0.15–0.43	0	4		
Calculations	CBC	0.02	0.14	-0.02–0.06	0	1	2.04 (*FWelch)*	0.014
	ABN	0.08	0.33	0.00–0.16	0	2		
	Total	0.06	0.27	0.01–0.11	0	2		
Concepts	CBC	0.93	0.25	0.85–1.00	0	1	5.85*	0.050
	ABN	1.02	0.17	0.98–1.07	1	2		
	Total	0.99	0.21	0.01–0.95	0	1		
TEMA-3 Total score	CBC	45	24.27	22.23–26.30	5	43	52.26**	0.320
	ABN	68	33.60	31.98–35.22	17	47		
	Total	113	29.89	28.37–31.40	5	47		


**p < 0.01, *p < 0.05.

Since five different schools participated in this study, a cross-school analysis has been carried out. This statistical analysis generated two categories of schools according to the Autonomous Community to which they belong: Andalusia and Madrid. The purpose was to explore whether there were differences. Comparing the average scores in informal and formal thinking, in each age group analyzed, independently of the instructional method, few statistically significant differences in most of the dimensions explored were found. For 4 year-old groups no statistically differences were found in informal reasoning (counting, comparing, informal calculations, informal concepts), either formal reasoning (conventions, number facts, formal concepts, comparing). For the 5 year-old group no statistically significant differences were found in comparing, number facts and formal concepts. However, differences were found for 5 year-old group in informal reasoning [*F*_(1,111)_ = 19.011, *p* < 0.05, η^2^ = 0.146], and the following subtests of this component: counting [*F*_(1,111)_ = 18.249, *p* < 0.05, η^2^ = 0.141]; informal calculations [*F*_(1,111)_ = 13.616, *p* < 0.05, η^2^ = 0.109], and informal concepts [*F*_(1,111)_ = 20.331, *p* < 0.05, η^2^ = 0.155]. Similarly, differences were found in formal reasoning [*F*_(1,111)_ = 14.67, *p* < 0.05, η^2^ = 0.117]: conventions [*F*_(1,111)_ = 13.565, *p* > 0.05, η^2^ = 0.109], and formal calculations [*F*_(1,111)_ = 1.954, *p* > 0.05, η^2^ = 0.108].

Statistically significant differences were found in 9 out of 10 dimensions compared (*p* < 0.01). In particular, counting dimension, which is part of the development of informal reasoning, a large effect size was found. In addition, significant differences and a large effect size were found of convention dimensions, which is part of development of formal reasoning.

In order to support that instructional approach generated a positive interaction cross-age effect, an additional statistical analyzes were carried out. This effect reproduces time effect learning with the instruction methodology (ABN or CBC), by observing the informal and formal mathematical thinking tasks data analyzed.

A significant disordinal interaction between the teaching method and students age was found, regarding the level of mathematical informal thinking: The methods’ effect was not the same for each age students group, but the difference was always for the ABN group [*F*_(1,220)_ = 10.68; *p* < 0.05, η^2^ = 0.046]. Similarly, statistical differences were found considering the learning method [*F*_(1,220)_ = 37.11; *p* < 0.05, η^2^ = 0.144]; and by age group [*F*_(1,220)_ = 60.34; *p* < 0.05, η^2^ = 0.215]. These main effects indicated that ABN method’ students achieved higher than CBC method’ students. Furthermore, 5-year-old students group achieved better in math informal thinking than their 4 years peers; *(a) Mathematical formal thinking dimension comparison*. Regarding the math formal thinking dimension, a significant interaction effect between instructional method and age group was found [*F*_(1,220)_ = 16.39; *p* < 0.05, η^2^ = 0.069]. In the same way, differences were found by learning methodology (ABN or CBC) [*F*_(1,220)_ = 28.94; *p* < 0.05, η^2^ = 0.116]; and by age group [*F*_(1,220)_ = 40.01; *p* < 0.05, η^2^ = 0.154]. Concerning the dimensions that conform the math formal thinking, interaction effect was found in conventionalism [*F*_(1,220)_ = 17.99; *p* < 0.05, η^2^ = 0.076]; but none in numerical facts [*F*_(1,220)_ = 3.09; *p* > 0.05, η^2^ = 0.014]. Main effects were found according to the ABN or CBC method [*F*_(1,220)_ = 6.96; *p* < 0.05, η^2^ = 0.031]; and by age group [*F*_(1,220)_ = 8.50; *p* < 0.05, η^2^ = 0.037]. None interaction effects were found in formal calculus [*F*_(1,220)_ = 1.46; *p* > 0.05, η^2^ = 0.007]; although the main effect attributable to the age group was found [*F*_(1,220)_ = 4.10; *p* < 0.05, η^2^ = 0.018], but not by method [*F*_(1,220)_ = 1.46; *p* > 0.05, η^2^ = 0.007], neither in formal mathematical thinking [*F*_(1,220)_ = 2.58; *p* > 0.05, η^2^ = 0.012]. A main effect attributable to the age group was observed [*F*_(1,220)_ = 3.94; *p* < 0.05, η^2^ = 0.018], rather than by the method [*F*_(1,220)_ = 0.81; *p* > 0.05, η^2^ = 0.004]; *(b) TEMA-3 score comparison*. Regarding the TEMA 3 direct scores, a significant interaction between mathematics learning method (ABN or CBC) and the students’ age [*F*_(1,220)_ = 13.05; *p* < 0.05, η^2^ = 0.056] was found between. Significant differences were also observed by the learning method [*F*_(1,220)_ = 37.92; *p* < 0.05, η^2^ = 0.147]; and by age group [*F*_(1,220)_ = 59.44; *p* < 0.05, η^2^ = 0.213]. These main effects suggest that participants who learned mathematics with the ABN approach achieved higher results than students who learned with the CBC method. Similarly, 5-year-old students achieved higher average scores in informal mathematical thinking than those in 4 year-old group.

For numerical estimation tasks, statistically significant differences were found in 5-year-old group. Specifically, when comparing student averages regarding the ability to estimate numbers, significant differences were found for the ABN-group, with a small effect size (**Table 4**).

**Table 4:** Descriptive and inferential analyses results for the numerical estimation task for CBC and ABN groups.

Course	Method						*F*	η^2^
			
	CBC			ABN				
			
	*M*	*SD*	*N*	*M*	*SD*	*N*		
4 year-old	2.46	1.80	37	3.22	1.96	74	3.871	0.034
5 year-old	2.58	2.01	45	3.94	1.99	68	12.50**	0.101
Total	2.52	1.91	82	3.56	2.05	142	14.43**	0.061


**p < 0.01.

## Discussion

The previous results support the hypothesis about the positive impact of the ABN method on the dimensions that make up formal and informal reasoning. Namely, the participants who were under the ABN approach obtained significantly higher results than the CBC approach. Thus, it can be maintained that the ABN method provides an integrated perspective, based on the significant learning of the decimal counting system, as well as providing complete understanding of basic math processes and their properties (Martínez-Montero, 2010, 2011; Martínez-Montero and Sánchez, 2011). Even so, results indicate that this differential gain is substantial for the 5-year-old group, where statistically significant differences were found in all formal and informal mathematical reasoning dimensions. For the 4-year-old children, informal calculations and informal concepts dimensions were found statistically significant.

A potential explanation for these results is that children in the 5-year-old group have received 2 years of systematic formal instruction in mathematics, which has included both ABN and CBC approaches. Therefore, the ABN group students showed a higher ability to successfully solve the tasks that compose each of the dimensions associated with formal and informal reasoning, assessed by TEMA-3 test, because the test scores provide a standardized measure of early arithmetic performance (Núñez and Lozano, 2009; Ryoo et al., 2015). These results are relevant, because the tested skills for informal mathematical reasoning, such as the ability to pay selective attention to numbers contribute to the development of the number sense. This is one of the main predictors of arithmetic performance. In addition, this type of number acuity reinforces mathematical achievement in early childhood, even though the influence of non-numerical characteristics significantly decreases when children developmental progresses. Even so, the ability to pay selective attention remains as a determining factor for decision-making at all ages (Starr et al., 2017).

Similarly, the ability to compare properly and quickly numerical magnitudes, was a predictor of achievement in mathematics, independent of age, intellectual capacity, and number identification speed (De Smedt et al., 2009; Fazio et al., 2014). Several studies have found that babies are able to selectively pay attention to numbers and size, which could be considered to be the basis for the development of number-sense (Cantrell and Smith, 2013; Mou and vanMarle, 2014; Szkudlarek and Brannon, 2017). Furthermore, there is evidence that number representations, even in adulthood, are influenced by non-numerical properties, such as the size of stimuli, for example (Defever et al., 2013; Fuhs and Mcneil, 2013; Gilmore et al., 2013; Szucs et al., 2013).

Likewise, there is significant evidence of the relationship between processing of cardinality and number seriation and arithmetic achievement. Namely, this is found in conditions involving easy multiplication, addition and subtraction tasks (De Smedt et al., 2013; Lyons et al., 2014; Chu et al., 2015; Vogel et al., 2015). Therefore, to improve all these capacities is optimistic, because it provides a better understanding of the cognitive architecture underlying achievement in early childhood education in mathematics, and the impact of different approaches for teaching and learning (Lo et al., 2017).

In summary, in the case of the 5-year-old group, significant differences were verified for the total number of dimensions that make up informal reasoning. In the case of the 4-year-old group, statistically significant differences for the ABN group were found just in calculation and informal concepts dimensions. Consequently, no significant differences were found for counting and comparison of quantities dimensions for this group. These results can be explained because ABN method produces differences achievement for the 4-year-old group for more complex informal tasks; in which students need not only knowledge of numbers, but also the management and application of resolution strategies.

With respect to the second hypothesis, for the 5-year-old group, the boys and girls under the ABN method showed a better performance on the direct score associated with formal reasoning than their peers under the CBC methodology did. Formal reasoning is a very relevant dimension of children’s mathematical reasoning, since it implies knowledge and skills, such as the conventions of reading and writing of quantities, the command of number facts, and formal calculation. In this sense, our findings provided relevance to the accuracy of formal procedures and to the basic concepts of the decimal system, such as space value and equivalences between different orders of magnitude. Even though the ABN method has been recently incorporated in Spanish school curricula, these findings coincide with other research previously conducted about the ABN method (Adamuz-Povedano and Bracho-López, 2014; Bracho-López et al., 2014b; Aragón et al., 2017a,b).

Considering the convention dimensions, the existence of errors in the literacy of the quantity for single items may indicate that certain rules have not been fully learned. This finding becomes clearer when other variables are controlled, such as the educational level of students’ parents and their previous levels of literacy. The ABN method contributes in making these operations automatic. In addition, the results obtained in this research are consistent with a previous study with primary school students. Bracho-López et al. (2014b) showed that a student’s following the ABN method got significant differences compared to their peers under the CBC approach in tasks involving the decimal system and number facts. Likewise, Moore et al. (2016) demonstrated that the cardinal knowledge exhibited by preschoolers, as well as their competence in manipulation of quantities associated with symbolic numerals, predicted higher flexibility in processing of magnitudes and in academic performance in the future. In this respect, the ABN group obtained significantly higher results in the domains of number facts and calculation when compared with the CBC group.

The significance of these findings lies in the fact that as children progress in a formal instruction, they are expected to begin using algorithms that synthesize sequences of unambiguous instructions to obtain a required result; algorithms that are also assumed to contribute to the basic understanding of mathematical concepts (Levitin, 2015). Hence, finding a better performance in these tasks in the ABN group evidences the strengthening and earlier consolidation of these type of procedural logic operations of abstract nature. In the same way, it is very important to achieve proficiency of number facts, since they are one of the central goals for the mathematics teaching in early education. Namely, students in their 1st years of formal education must be able to remember and provide fast responses to the calculations involved in basic tasks. Domain number facts facilitates and speeds up these processes, leaving space and opportunity to better understanding. It is worth to mention that a good command of this skill does not only involve memory, but is also linked to the application of previously stored rules, which allows easier access to the processing of symbolic magnitudes (De Smedt et al., 2013).

Finally, with respect to formal calculation, the ABN students also obtained better results than their peers following the CBC methodology. It is important to remember that performing formal calculations entails a command of the decimal number system, counting strategies, and knowledge of number facts. In the case of the ABN method, algorithmic operations are part of learning activities and contribute to meaning making. These two facts contribute to a better predisposition and attitude toward to complete the task, which has a favorable impact in the conceptual knowledge that children incorporate and bring into play when making strategic decisions for the resolution of problems (Robinson and Dubé, 2012).

The third hypothesis of this research focused around of significant differences between both approaches concerning numerical estimation in the number-line tasks. The results showed significant differences for the 5-year-old ABN group. According to a previous study, this advantage was also at the age of 6 years (Aragón et al., 2017b). The importance of this result lies in the fact that estimation skills contribute to a good arithmetic competence, since the proficiency in these tasks involves capacity to provide meaning to the magnitude of numbers in a number-line (Laski and Siegler, 2007). However, numerical estimation tasks stand out as one of the most complex numerical activities for 5-year-old students (Araújo et al., 2014). As students progress in a formal instruction, they make fewer errors and improve their accuracy (Siegler and Opfer, 2003). Therefore, an adequate training in basic concepts underling numerical estimation skills (as the ABN method does), contributes to improve this ability. Estimation is a relevant part of the prerequisites of early arithmetic skills and contributes to a good performance in mathematics in later stages of formal instruction. Concerning this matter, Watts et al. (2014) evaluated early arithmetic variables in early childhood education and confirmed that early arithmetic competences were predictors for achievement in primary, and also secondary education.

In short, the impact of the mathematical structure developed by the ABN method leads to a better understanding of several essential operations of Algebra (roots, powers, functions, for example). This is because algebra is a discipline in close conceptual relationship with arithmetic. However, in the case of Algebra, the processes and concepts involve a bigger capacity for abstraction. This capacity could be promoted through higher skills for arithmetic representation and could lay the foundations for algebraic representation (Humberstone and Reeve, 2018). Furthermore, this relationship could also explain the higher levels of achievement exhibited by the ABN children in formal and informal reasoning dimensions assessed.

Finally, it should be considered that the differences between the ABN and CBC methodologies are not limited to the mere use of a specific type of algorithmic strategy. The differences between the two approaches lies in understanding the mathematical structure underlying arithmetic operations, which is a critical factor for the development of many others mathematical skills (National Governors Association Center for Best Practices and Council of Chief State School Officers, 2010). From this point of view, we believe that the ABN method defines the processes for arithmetic operations by using methods of decomposition and tasks with quantities. It allows a better understanding of the underlying mathematical structure, as well as better predisposition and basis for the understanding of problems of an additive and multiplicative nature. It is well stablished that additive and multiplicative tasks share some concepts such as identity, negation, commutativity, equivalence, reversal, and associativity (Robinson et al., 2018). They are key for building the formal arithmetic knowledge in the algebra constructing phases.

### Study Limitations and Future Perspectives

Since this research was not an experimental time-series design, we are not able to attribute conclusively the difference in formal and informal mathematical reasoning to the instructional approaches used. Even so, the control tests used established that the qualifications of the ABN and CBC groups compared equivalent. Therefore, we can infer that the differences found can be function of the instructional approach, given that the immersion time of both groups corresponds to the same number of school years.

It should also be mentioned that the effect of the teacher instruction, in terms of years of service, initial teacher training approaches, and/or gender, as well as the students themselves, are also limitations that could have had an effect on the differential results observed, in the sense of mitigating or enhancing the observed differences.

Therefore, for future research and in order to have control over the sources challenging internal and external validity, a quasi-experimental longitudinal study is proposed. We believe that this type of design will strengthen the hypothesis about the favorable impact of the ABN instructional approach on informal and formal mathematical reasoning and it will provide a more robust basis to prove the methods’ positive effect on performance in the arithmetic development. Finally, we also deem that the effect of the ABN approach could be compared to other types of flexible or innovative approaches in the field of early mathematics teaching, in order to provide more support to the instructional potential of the ABN approach.

## Ethics Statement

This study was carried out in accordance with the recommendations of the Bioethics Committee of University of Cádiz, available at http://www.uca.es/recursos/doc/Unidades/normativa/investigacion/122607032_151201413525.pdf. It was also carried out in accordance with the National Commission for Scientific and Technological Research (CONICYT, Chile) http://www.conicyt.cl/fondecyt/category/estudios-y-documentos/bioetica/. The protocol was approved by the Bioethics Committee of the three participant universities. All subjects gave written informed consent in accordance with the Declaration of Helsinki and Singapore Statement. http://www.conicyt.cl/fondecyt/files/2017/06/Singapore_Statement.pdf.

## Author Contributions

GC statistical analysis, literature review, and writing the final version of the manuscript. EA application of assessment tools in educational establishments and statistical analysis of results. CP theoretical discussion of instructional methods, fundamentals of the ABN methodology and contextualization of statistical results according the ABN focus. JN theoretical discussion of Early Mathematical competences, analysis of comparable results in literature, and review of the draft version of the manuscript. MA theoretical discussion of psychometric instruments applied. Application of assessment tools in educational establishments, responsible for the ethical consent of the participants.

## Conflict of Interest Statement

The authors declare that the research was conducted in the absence of any commercial or financial relationships that could be construed as a potential conflict of interest.
